# Analysis of bi-atrial function using CMR feature tracking and long-axis shortening approaches in patients with diastolic dysfunction and atrial fibrillation

**DOI:** 10.1007/s00330-023-09663-4

**Published:** 2023-05-05

**Authors:** Dominik P. Guensch, Shagana Kuganathan, Christoph D. Utz, Mario D. Neuenschwander, Leonard Grob, Philipp Becker, Salome Oeri, Adrian T. Huber, Martina Boscolo Berto, Giancarlo Spano, Christoph Gräni, Matthias G. Friedrich, Balthasar Eberle, Kady Fischer

**Affiliations:** 1 Department of Anaesthesiology and Pain Medicine, Inselspital, Bern University Hospital, University of Bern, Bern, Switzerland; 2 Department of Diagnostic, Interventional and Paediatric Radiology, Inselspital, Bern University Hospital, University of Bern, Bern, Switzerland; 3 Department of Cardiology, Inselspital, Bern University Hospital, University of Bern, Bern, Switzerland; 4 Department of Medicine, McGill University, Montreal, QC Canada; 5 Department of Radiology, McGill University, Montreal, QC Canada

**Keywords:** Atrial function, Atrial fibrillation, Heart failure, Magnetic resonance imaging

## Abstract

**Objectives:**

Atrial function can be assessed using advancing cardiovascular magnetic resonance (CMR) post-processing methods: atrial feature tracking (FT) strain analysis or a long-axis shortening (LAS) technique. This study aimed to first compare the two FT and LAS techniques in healthy individuals and cardiovascular patients and then investigated how left (LA) and right atrial (RA) measurements are related to the severity of diastolic dysfunction or atrial fibrillation.

**Methods:**

Sixty healthy controls and 90 cardiovascular disease patients with coronary artery disease, heart failure, or atrial fibrillation, underwent CMR. LA and RA were analyzed for standard volumetry as well as for myocardial deformation using FT and LAS for the different functional phases (reservoir, conduit, booster). Additionally, ventricular shortening and valve excursion measurements were assessed with the LAS module.

**Results:**

The measurements for each of the LA and RA phases were correlated (*p* < 0.05) between the two approaches, with the highest correlation coefficients occurring in the reservoir phase (LA: *r* = 0.83, *p* < 0.01, RA: *r* = 0.66, *p* < 0.01). Both methods demonstrated reduced LA (FT: 26 ± 13% vs 48 ± 12%, LAS: 25 ± 11% vs 42 ± 8%, *p* < 0.01) and RA reservoir function (FT: 28 ± 15% vs 42 ± 15%, LAS: 27 ± 12% vs 42 ± 10%, *p* < 0.01) in patients compared to controls. Atrial LAS and FT decreased with diastolic dysfunction and atrial fibrillation. This mirrored ventricular dysfunction measurements.

**Conclusion:**

Similar results were generated for bi-atrial function measurements between two CMR post-processing approaches of FT and LAS. Moreover, these methods allowed for the assessment of incremental deterioration of LA and RA function with increasing left ventricular diastolic dysfunction and atrial fibrillation.

**Clinical summary statement:**

A CMR-based analysis of bi-atrial strain or shortening discriminates patients with early-stage diastolic dysfunction prior to the presence of compromised atrial and ventricular ejection fractions that occur with late-stage diastolic dysfunction and atrial fibrillation.

**Key Points:**

*• Assessing right and left atrial function with CMR feature tracking or long-axis shortening techniques yields similar measurements and could potentially be used interchangeably based on the software capabilities of individual sites.*

*• Atrial deformation and/or long-axis shortening allow for early detection of subtle atrial myopathy in diastolic dysfunction, even when atrial enlargement is not yet apparent.*

*• Using a CMR-based analysis to understand the individual atrial-ventricular interaction in addition to tissue characteristics allows for a comprehensive interrogation of all four heart chambers. In patients, this could add clinically meaningful information and potentially allow for optimal therapies to be chosen to better target the dysfunction.*

**Graphical abstract:**

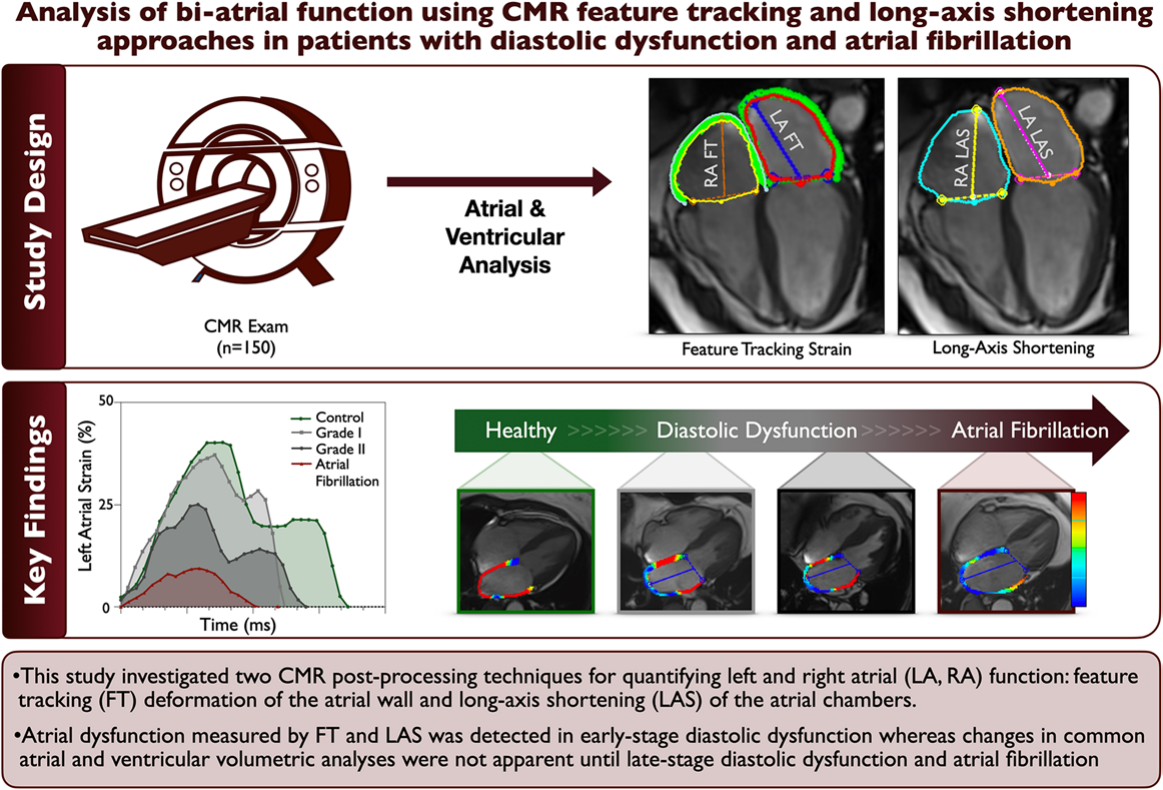

**Supplementary Information:**

The online version contains supplementary material available at 10.1007/s00330-023-09663-4.

## Introduction

Left ventricular (LV) diastolic dysfunction is a highly prevalent condition that is associated with ventricular and atrial remodeling and has strong associations with heart failure and atrial fibrillation (AF) [1, 2]. LA volume indices are included in recommendations for multi-parametric classification of diastolic dysfunction [3]. With progressive diastolic dysfunction, the LA enlarges, and with time, atrial fibrosis ensues. A dysfunctional or non-compliant LV not only increases left atrial (LA) pressures but can also lead to right ventricular (RV) and atrial (RA) impairment, and symptoms of heart failure [4, 5]. Thus, imaging of cardiac function ideally includes all four chambers.

Functional assessment is commonly performed using dimensions or volumes. However, volumetric analysis either requires complete coverage of the chamber, which is time-consuming, or relies on geometric assumptions when using fewer image planes. A different approach is through deformation analysis, or strain imaging, which can be performed for the atria using echocardiography, computed tomography, or cardiovascular magnetic resonance (CMR) [6–8]. Feature tracking (FT) analysis is a post-processing technique that measures the deformation of the atrial wall. Atrial FT has been associated with both other markers of atrial dysfunction such as atrial fibrosis [9], and future outcomes including atrial fibrillation and hospitalization for heart failure [10, 11]. FT however relies on tracking throughout the cardiac cycle, so reproducibility can be reduced in disease cohorts [12, 13]. A second, more recent analysis technique is using rapid semi-automated methods to measure long-axis shortening (LAS) of the atrial chambers and valve excursion, rather than the deformation of the atrial walls [14]. This technique is based on reference points at the valves, atrial roof, and ventricular apex to provide information on all four chambers within the same analysis. Data on CMR atrial strain are still emerging and especially for LAS post-processing techniques, information is currently scarce.

Thus, this study aimed to investigate and compare atrial FT and LAS CMR analysis approaches for atrial function in healthy controls and how they compare to cardiovascular disease patients with diastolic dysfunction and atrial fibrillation (AF).

## Methods

### Dataset inclusion

Sixty healthy controls and ninety cardiovascular disease patients with either heart failure [15], paroxysmal or persistent AF, and/or obstructive coronary artery disease were included in the analysis (Supplemental Fig. 1). From clinical echocardiography exams performed within 28 days of the CMR exam, the grade of diastolic dysfunction was acquired from the patients’ clinical diagnosis written in the report and further verified with diastolic dysfunction guidelines based on the reported echocardiographic measurements[3]. Patients were then grouped into one of four categories: no diastolic dysfunction (grade 0), diastolic dysfunction grade I, a combined group of diastolic dysfunction grade II or III, or as having AF. Healthy controls were defined as subjects 18 to 70 years of age without cardiovascular or respiratory disease or medication that would impact these systems. All participants had provided written consent for secondary use of data. This protocol was reviewed and approved by the ethics committee of the canton of Bern. (MACDAVD study, approval number 2020_01258).

### CMR image acquisition

Images were obtained with a 3.0-Tesla clinical scanner (Siemens Prisma or Skyra, Siemens Healthineers). Retrospective-gated cine images with 25 − 30 phases were acquired in short-axis and long-axis views orientated to the left ventricle. In the case of varying RR intervals, gating was changed to prospective triggering at the discretion of the imaging technician. T1 maps (5(3)3-modified look-locker inversion recovery) were acquired in three short-axis views. In cardiovascular patients, 0.1–0.15 mmol/kg of contrast agent (Gadovist™, Bayer AG), was administered and T1 maps were reacquired post-contrast for the assessment of extracellular volume (ECV) while late gadolinium enhancement (LGE) images were acquired for assessment of focal scar[16].

### CMR image analysis

All images underwent a second level of coding to blind the reader from the originating study and site, disease status, and other variables. Analysis was performed with cvi^42^ (version 5.12–5.13, Circle Cardiovascular Imaging). Ventricular parameters were measured as previously described [16, 17].

#### Atrial analysis feature tracking

**Fig. 1:**
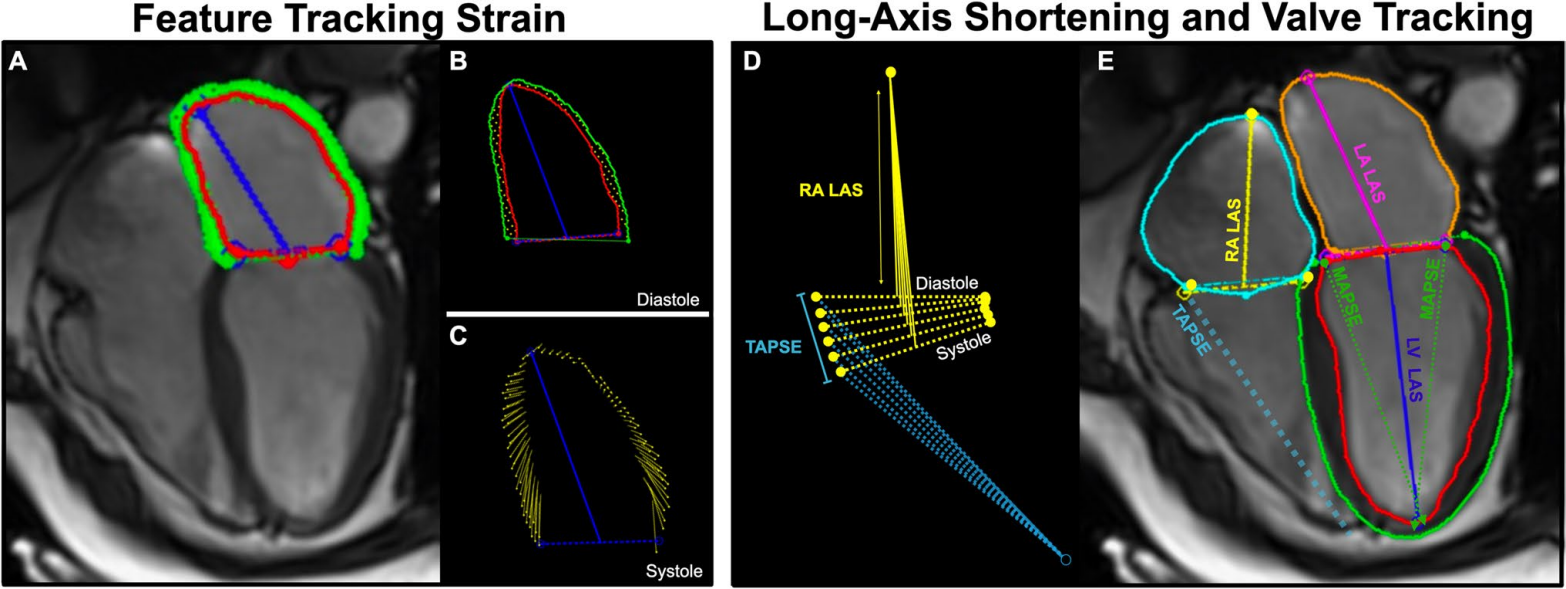
Mono- and bi-planar atrial analysis methods. Feature tracking (FT) analysis (**A**) is performed starting with atrial contours on the phase of ventricular end-diastole (**B**) and measures longitudinal extending of the atrial wall muscle fibers (**C**). On the other hand, long-axis shortening techniques quantify the change in the extent of the atrial chambers measured from the atrial roof to the center of the valve, an example of the right heart is shown in **D**. Additionally two-dimensional atrial dimensions, volumes, and ventricular measurements are acquired (**E**). LA: left atrial, LAS: long-axis shortening, MAPSE: mitral annular planar systolic excursion, RA: right atrial, TAPSE: tricuspid annular planar systolic excursion

Atrial strain analysis was performed using FT. Epicardial and endocardial contours were placed on LA on both the four-chamber (4CH) and two-chamber (2CH) views, and on RA of the 4CH view on a single phase at ventricular end-diastole. For both atria, the free wall and the atrial septum were included in the measurements (Fig. 1). Endocardial longitudinal strain and strain rates were then calculated for the cardiac cycle and measurements were categorized into the three atrial phases (depicted in Fig. 6);

1. The *reservoir phase* indicating the filling phase of the atria from minimal to maximal volume, strain, or extent, occurring during ventricular systole. This is also known as the atrial peak strain and the change in volume over this phase is used to calculate the atrial ejection fraction.
2. The *conduit phase* comprised of passive atrial emptying, occurring during early ventricular diastole
3. The *booster phase* for active atrial emptying, also known as the atrial kick

#### Atrial volumetry and long-axis shortening

Using the 2CH and 4CH cines, contours defining the LA and RA endocardium, LV endo- and epicardium, and valvular junctions were drawn automatically using a machine learning algorithm of the module for each phase of the cardiac cycle. Each image was reviewed by the reader and adjusted manually if required. Biplanar atrial areas and volumes were calculated. LA and RA long-axis shortening (LAS) were calculated from the longitudinal shortening of the atria from the atrial roof to the center of the atrioventricular valve (Fig. 1D) for each of the three atrial phases. Additional parameters were determined including left ventricular epicardial long-axis strain (LV-LAS%) from the biplanar view. Valve displacement was measured for tricuspid annular planar systolic excursion (TAPSE) from the 4CH cine. Mitral annular planar systolic excursion (MAPSE) was averaged from four sites (inferior, anterior, lateral, and septal) using both the 2CH and 4CH views.

### Statistical analysis

First to compare the atrial analysis techniques, measurements from the atrial biplanar volumetric analysis, FT strain and LAS, were compared using correlation analysis. Agreement between techniques was further tested with the Bland-Altman analysis. Findings were first compared between the healthy controls and the entire patient group using a general linear model including age as a covariate. To further account for age differences between patients and healthy controls, a subgroup of healthy controls consisting of only the oldest tertile was defined. This older control subgroup was then compared to the diastolic dysfunction patient subgroups using ANOVA and post hoc analysis accounting for multiple comparisons. Logistic regression was used to quantify the area under the curve (AUC) to investigate the ability of atrial measurements to discriminate the patient subgroups. Statistical significance was defined with a two-sided* p* value of  < 0.05. GraphPad Prism version 8.0 (GraphPad Software, and SPSS Statistics 26 (IBM) were used for statistical analysis.

## Results

### Patient characteristics and image inclusion

Detailed participant characteristics are provided in Supplemental Table 1. Of the 90 patients, 24 (27%) patients were classified with diastolic dysfunction grade I, 20 (22%) patients with diastolic dysfunction grade II/III, and 20 (22%) patients had atrial fibrillation (paroxysmal *n* = 12, persistent *n* = 8). The remainder had normal diastolic dysfunction or were not classified. Heart failure was diagnosed in *n* = 53 (59%) of the total cohort and was frequent in the diastolic dysfunction grade I (*n* = 17, 71%), grade II/III (*n* = 18, 90%), and AF subgroups (*n* = 13, 65%). Heart failure with preserved ejection fraction (HFpEF) was more common than heart failure with reduced ejection fraction (HFrEF) in both diastolic dysfunction grade I (HFpEF = 71% vs HFrEF = 29%) and diastolic dysfunction grade II/III (HFpEF = 94% vs HFrEF = 6%). The opposite was seen in patients with AF with a higher prevalence of HFrEF (HFpEF = 37% vs HFrEF = 69%). None of the patients with normal diastolic function had heart failure (*n* = 0, 0%).

**Fig. 2:**
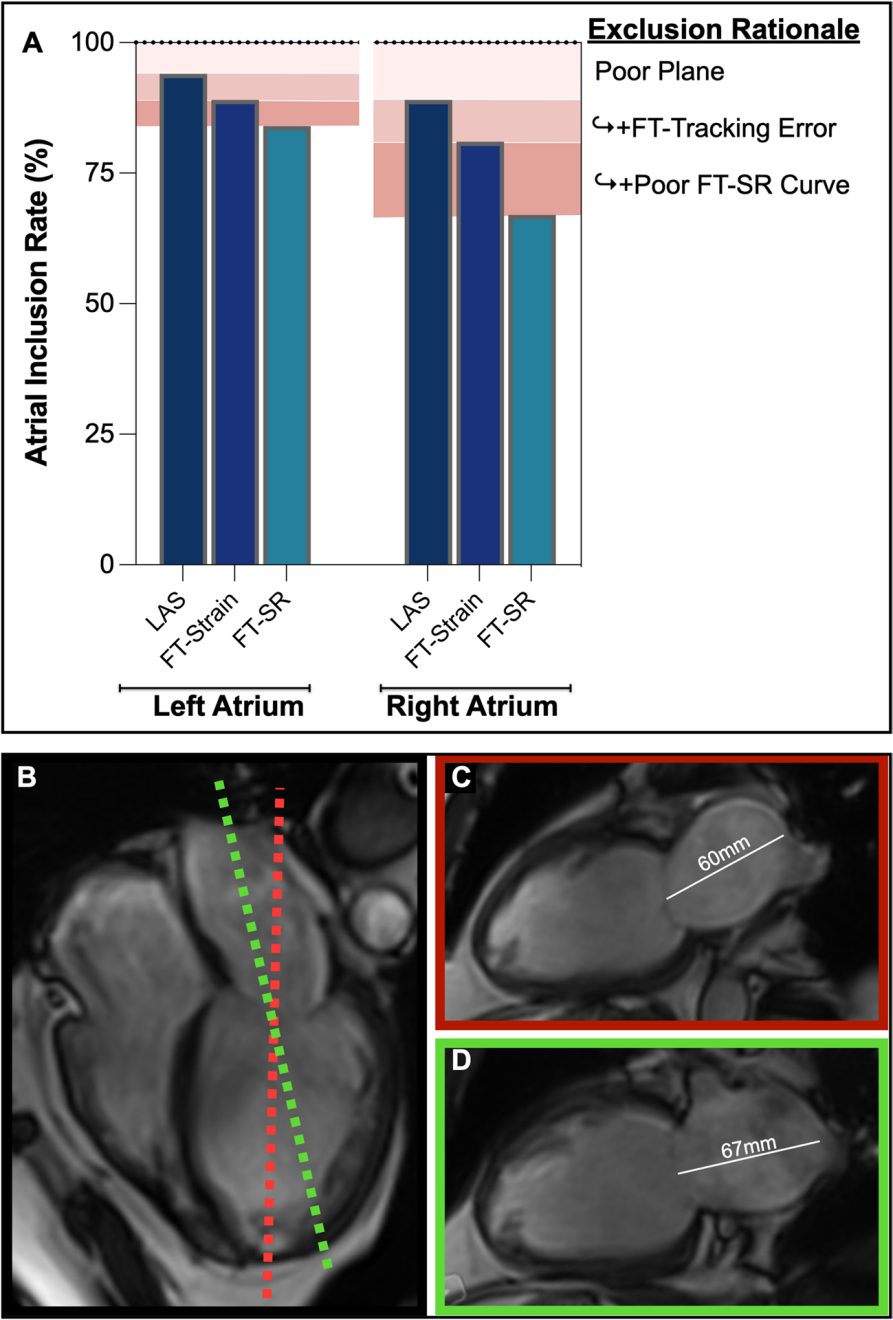
Atrial planes. The proportion of atrial images included in the analysis (**A**) indicates that while 6% of left atrial images and 11% of right atrial images were excluded for a poor plane position, all remaining images could be used for long-axis shortening (LAS) analysis, while additional images were excluded from feature tracking (FT) analysis due to tracking errors and poor strain rate (SR) curves. The lower panel from a patient with atrial fibrillation demonstrates that while the plane indicated in panel **C** (red) is the ideal location for ventricular analysis cutting perpendicular through the mitral valve and ventricular apex, this can lead to foreshortening of the left atrium (**B**), while panel **D** (green) depicts the true atrial length. However, image acquisition is typically localized off the ventricle leading to the proportion of datasets excluded due to the off-angle plane of the atria

The left ventricular analysis could be acquired from all 150 datasets. Concerning the LA, nine patients were excluded from the analysis because 2CH and 4CH views were off the plane with foreshortening, in which both the atrial roof and the middle of the valve were not visible. Thus, 94% of the cines were available for analysis. All remaining images could be analyzed for volumetric and rapid LAS atrial analysis, with 84% eventually being usable for FT analysis after excluding another seven for tracking errors. Similarly for the RA, sixteen images were excluded due to plane position, thus 89% were usable for volumetric and atrial LAS measurements which could be performed on all appropriate planes, with 81% eventually being usable for the FT strain analysis (Fig. 2, Supplemental Fig. 1).

### Comparison between atrial analysis techniques

**Fig. 3:**
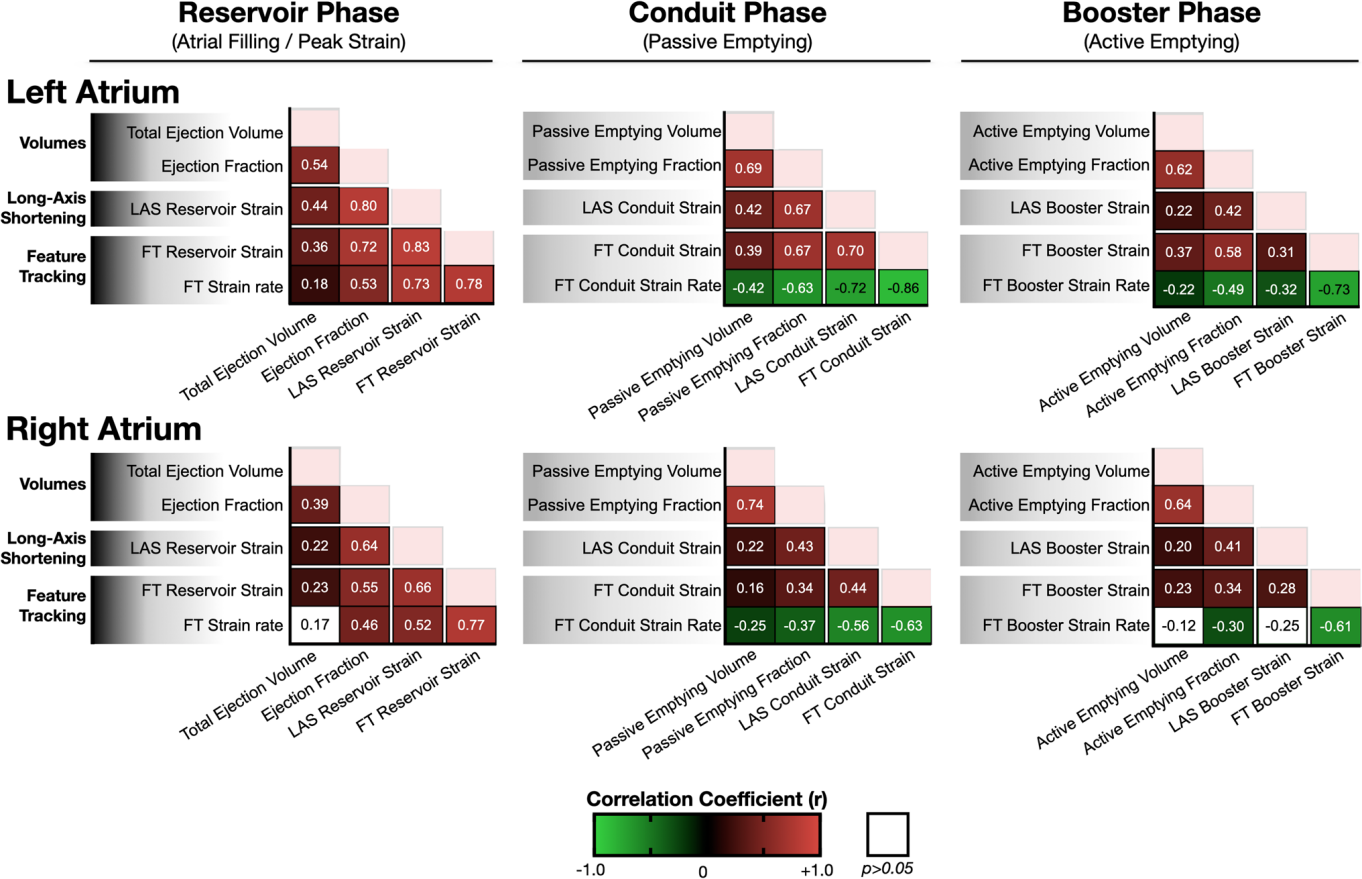
Atrial correlograms. Correlograms depict linear relationships between atrial volumetry, long-axis strain, and feature tracking measurements for the three phases of atrial function. Significant correlations (*p* < 0.05) are color-coded by the correlation coefficient, and non-significant correlations are depicted by white squares. FT: Feature tracking, LAS: Long-axis strain

Correlograms in Fig. 3 demonstrate that the highest linear correlation coefficients were found between FT strain and LAS, while associations between FT strain rate and atrial volumetry were weaker. In particular, when focusing on the reservoir (peak) strain of the LA, a good correlation of *r* = 0.83, *p* < 0.01 was observed between the FT and LAS measurements for all participants. For this comparison, analysis from the Bland-Altman yielded a non-significant bias of  − 3.0 ± 9.3% for the LAS measurements in comparison to FT (Supplemental Fig. 2, Supplemental Table 2). Even when performing this analysis for just patients with diastolic dysfunction (*r* = 0.75, *p* < 0.01) or AF (*r* = 0.88, *p* < 0.01), similar comparisons were observed between the two techniques (Supplemental Table 3). For the same comparison in the RA, a correlation of *r* = 0.66, *p* < 0.01 was observed with a bias of − 0.2 ± 12.5% when investigating all participants, with equal comparisons observed in the subsets of patients with diastolic dysfunction (*r* = 0.65, *p* < 0.01) and AF (*r* = 0.73, *p* < 0.01). Significant, but weaker linear relationships were observed for the conduit and booster atrial phases.

### Atrial function and severity of diastolic dysfunction

**Fig. 4:**
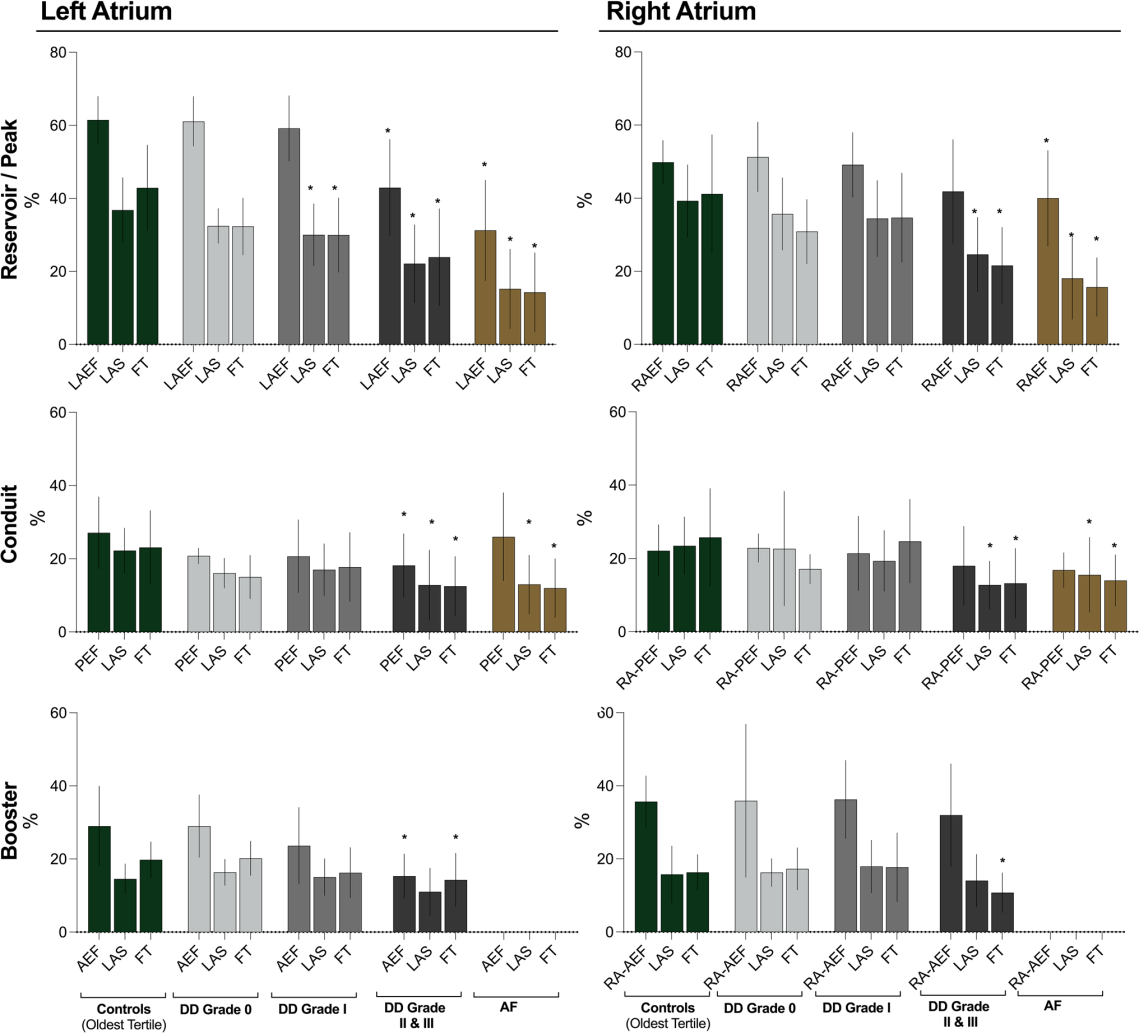
Atrial measurements by diastolic dysfunction. Mean ± SD measures are shown for volumetric fractions, along with long-axis shortening (LAS) and feature-tracking (FT) strain (Supplemental Tables 4–5). *p < 0.05 versus older controls accounting for multiple comparisons. AEF: active emptying fraction, AF: atrial fibrillation, DD: diastolic dysfunction, EF: ejection fraction, LA; left atrial, PEF: passive emptying fraction, RA: right atrial

**Fig. 5:**
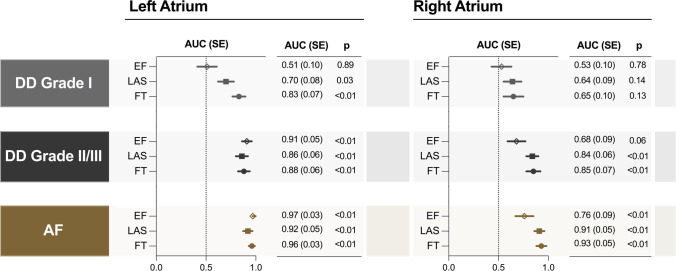
Atrial analysis discriminates patient subgroups. Area under the curve (AUC) and standard error (SE) demonstrate that long-axis shortening (LAS) and feature-tracking (FT) strain techniques can discriminate diastolic dysfunction (DD) grade I patients based on the left atrium from older controls, while left atrial ejection fraction (EF) only can discriminate patients with DD grade II/III or atrial fibrillation (AF). Using right atrial measurements, LAS and FT can discriminate DD grade II/III patients, while right atrial EF can only discriminate AF patients

**Fig. 6:**
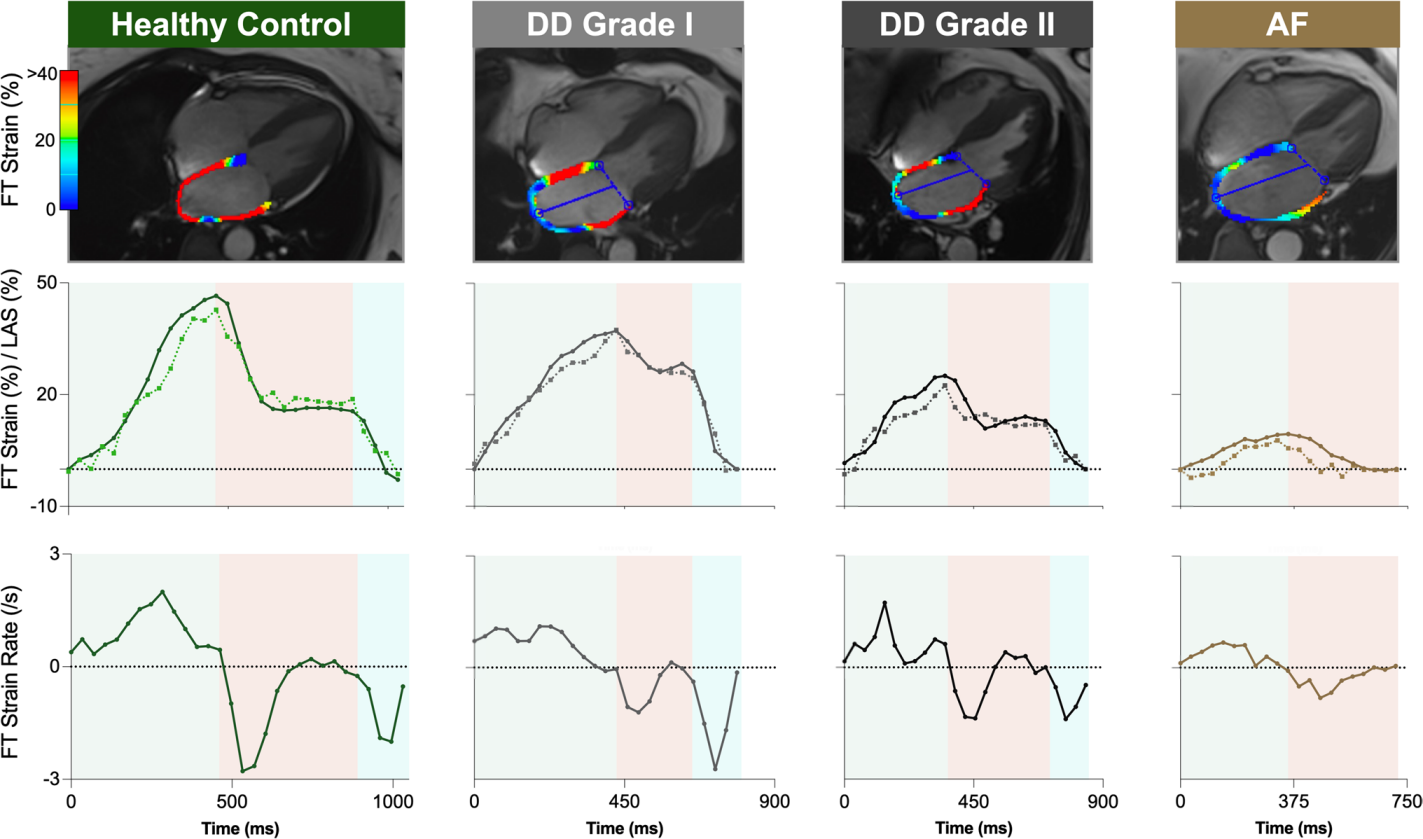
Case examples. Top: Feature tracking (FT) strain analysis of the left atrium at peak atrial strain. Middle: FT strain curves (solid line) and long-axis shortening (LAS, dotted lines), are plotted over the cardiac cycle, with the FT strain rate plotted on the bottom row. Green shading indicates the reservoir phase, red is the conduit phase and blue is the booster phase. AF: atrial fibrillation, DD: diastolic dysfunction

In comparison to the older control subgroup (FT: 43 ± 12%, LAS: 37 ± 9%), the patient subgroups with diastolic dysfunction (grade I: 30 ± 10% and 30 ± 8%, grade II/III: 24 ± 13% and 22 ± 11%, for FT and LAS respectively) or AF (FT: 14 ± 11% and LAS: 15 ± 11%) had a significantly reduced reservoir strain (*p* < 0.05). On the other hand, significant reductions in LA ejection fraction only occurred with the grade II/III diastolic dysfunction and AF subgroups (Fig. 4, Supplemental Table 4). Consequently, logistic regression showed that only FT and LAS measurements could discriminate patients with diastolic dysfunction grade I from older controls, while all techniques were able to discriminate the later grades of diastolic dysfunction and AF (Fig. 5). Reduced strain measurements in the conduit and booster phase were observed with diastolic dysfunction grade II/III by both FT and LAS. Patients with AF also showed a reduced conduit function, and no booster function was detected (Fig. 6).

With RA analysis, no significant findings were observed for either the RA reservoir or RA passive measurements in grade 0 or grade I patients in comparison to the older control group. Only diastolic dysfunction grade II/III and AF subgroups demonstrated attenuated atrial function measurements for these two phases (Supplemental Table 5). Similar to the LA, LAS and FT in the RA were able to discriminate patients in earlier subgroups than RA ejection fraction (Fig. 5).

### Rapid LAS ventricular and valve tracking

In comparison to the traditional ventricular measurements of ejection fraction which only showed attenuation in the AF subgroup, ventricular markers of LV-LAS, MAPSE, and TAPSE from the LAS and valve tracking module demonstrated attenuation with the diastolic dysfunction grades I, II/III, and AF subgroups (Table 1). LV tissue characterization mirrored these results, showing that native T1 in grade 0 was similar to the controls, grade I, II/III, and AF subgroups all had higher native T1.

**Table 1:** Ventricular measurements

	Controls	All patients	Controls oldest tertile	DD grade 0	DD grade I	DD grade II-III	AF
Function							
LV ejection fraction (%)	63 ± 7	57 ± 12	61 ± 6	60 ± 9	61 ± 12	55 ± 10	46 ± 10^*#*^
LV-LAS (%)	− 19.5 ± 2.0	− 14.9 ± 6.3*	− 20.0 ± 4.1	− 16.0 ± 4.4	− 15.6 ± 5.5^*#*^	− 13.5 ± 6.5^*#*^	− 11.5 ± 5.8^*#*^
MAPSE_average_ (mm)	17.1 ± 2.6	11.3 ± 3.6*	16.0 ± 2.6	13.1 ± 3.2	11.5 ± 3.1^*#*^	11.0 ± 2.9^*#*^	9.5 ± 5.1^*#*^
TAPSE (mm)	24.8 ± 4.4	16.9 ± 6.4*	23.5 ± 3.8	20.9 ± 5.4	18.7 ± 6.2^*#*^	15.9 ± 5.8^*#*^	11.5 ± 4.8^*#*^
LV tissue characterization							
Native T1 (ms)	1205 ± 35	1256 ± 55*	1210 + 43	1236 ± 73	1261 ± 53^*#*^	1284 ± 60^*#*^	1251 ± 45^*#*^
Extracellular volume (%)^☨^	-	30.3 ± 6.2	-	30.5 ± 10.6	29.8 ± 5.9	31.2 ± 6.7	29.1 ± 4.4
Percent of patients with ≥ 30% extracellular volume^☨^	-	27%	-	20%	27%	38%	19%
Percent of patients with LGE present☨	-	36%		80%	19%	44%	29%

Mean ± SD. *AF* atrial fibrillation, *DD* diastolic dysfunction, *FT* feature-tracking, *LAS* long-axis shortening, *LGE* late gadolinium enhancement, *LV* left ventricular, *MAPSE* mitral annular planar systolic excursion, *TAPSE* tricuspid annular planar systolic excursion

^*^p < 0.05 all patients vs control population statistically correcting for age

^#^*p* < 0.05 and oldest tertile of controls compared to cardiovascular patient subgroups accounting for multiple comparisons

**☨**contrast agent was not administered in controls; thus no statistical comparison was performed

## Discussion

Feature tracking (FT) and long-axis shortening (LAS) post processing analysis methods provided consistent results for left and right atrial measurements in a cohort of 150 controls and cardiovascular patients. Even in diastolic dysfunction or AF, these atrial analysis techniques were comparable. Moreover, both methods demonstrated reduced atrial movement in patients with diastolic dysfunction and atrial fibrillation. These reductions in atrial function measured by FT and LAS were able to discriminate patients with less severe stages of diastolic dysfunction whereas changes in common volumetric analyses including atrial ejection fraction did not appear until later stages.

### Atrial strain and longitudinal shortening in comparison to volumetry

These findings indicate that both FT-strain and LAS measures may allow for detecting atrial abnormalities at an early stage of disease, consistent with echocardiography investigations [1, 18, 19]. Speckle tracking techniques have shown that LA strain is abnormal when left atrial volume indices are within reference ranges in patients with diastolic dysfunction [20], and an independent publication reported that replacing left atrial volume index with atrial strain reduced the proportion of indeterminate cases of diastolic dysfunction [21].

The key advantage of the FT and LAS techniques in comparison to volumetry is that they can be analyzed using minimal planes. Mono-planar and bi-planar volumetric analysis is a calculated representation of the atrial volume based on lumen diameters and area assuming an ideal spheric geometry. For more accurate volumetric analyses, full-stack atrial imaging would be required. However, when using current segmented cine techniques these additional slices required for full coverage volume analysis add time to image acquisition and analysis; consequently, these new FT strain and LAS approaches offer a comprehensive analysis from a relatively parsimonious amount of data. In addition, these new approaches may be more sensitive than using biplanar volumetric assessment. Echocardiography studies have shown that LA peak strain is less load-dependent than atrial volumetry-derived function measurements [22]. However, 2D imaging still relies on the quality and location of image acquisition, and especially for the atria, acquisition axes are generally defined based on LV anatomy (Fig. 2).

### Left vs. right atrial dysfunction

Interestingly, although our RA results show the same trends as in the LA data, significant findings were typically observed only with more severe diastolic dysfunction or AF. For instance, attenuated RA reservoir FT strain and LAS were not observed until diastolic dysfunction grade II/III was present, and no technique could discriminate diastolic dysfunction grade I from the RA. Pathophysiological explanations may be based on backward failure from progressive left-heart disease, or direct interventricular interactions. Since we categorized our groups by grading LV diastolic dysfunction, it appears logical that increased LV diastolic pressures first induce LA enlargement and dysfunction. Over time, this can cause a backward failure chain reaction with pulmonary venous congestion, eventually exposing the RV and RA to increased filling pressures. Direct left-to-right interactions may occur since all heart chambers are confined in the same non-distensible pericardium. Here, the advantage of interrogating all four chambers is that the function of each atrial and ventricular chamber can be assessed individually, also during identical phases of respiratory cycling, providing insight into the individual’s pathway of heart failure development. For example, in a recent pulmonary hypertension study, different atrial-ventricular interactions were observed in patients with pulmonary arterial hypertension secondary to HFpEF when compared with patients without HFpEF [23]. It is important to evaluate both sides of the heart because RA enlargement compared to LA size is an independent predictor for mortality [24]. A simultaneous four-chamber investigation may thus indicate whether pathophysiology is driven by the left or right side. Although our investigation focuses on LV diastolic dysfunction, RV diastolic dysfunction should not be discounted.

### Atrio-ventricular interactions

Atrial measurements are influenced by the dynamics of the atrio-ventricular interaction. Atrial reservoir (peak) function is dependent on both atrial stiffness and ventricular contractility during systole. On the other hand, conduit function is dependent on both atrial contractility and early LV relaxation. The ventricle is likely to play a larger role in atrial function in the early development of diastolic dysfunction. As the ventricle with diastolic dysfunction gradually stiffens, the atrium distends owing to increased atrial pressure, which in turn maintains blood flow into the ventricle in early diastole. We observed the earliest atrial FT-strain and LAS dysfunction during the LA reservoir phase (diastolic dysfunction grade I). This is consistent with previous findings from an echocardiography study where LA reservoir strain showed better agreement with invasively measured filling pressures, a hallmark of diastolic dysfunction, than the echocardiography marker E/e’ [25].

The LAS analysis module applied in our analysis is also useful because it provides results for ventricular systolic function, i.e. TAPSE, MAPSE, and LV-LAS, which were analyzed simultaneously with the atria. In comparison to the LV ejection fraction, which was only abnormal in the AF group, ventricular LAS along with the valve tracking markers of MAPSE and TAPSE detected abnormal ventricular dysfunction as early as diastolic dysfunction grade I, in line with the atrial measures. A unique characteristic of CMR in comparison to echocardiography is that its findings can be linked with tissue characterization measurements, which also reflected increasing myocardial fibrosis with increasing grades of diastolic dysfunction indicating a stiff LV.

### Clinical potential

This comparative study of atrial function assessments using the FT and LAS techniques indicated similar output, especially for the reservoir phase. Because LAS analysis was not solely reliant on tracking algorithms and contours could be manually adjusted, more patients could be included in the LAS analysis when compared to FT assessment of the same images. Especially in the case of AF, all images could be assessed for LAS. FT requires a good visualization of the entire atrial walls throughout the cardiac cycle, which made it prone to more errors and a higher user-defined exclusion rate. As LAS analysis is a simple calculation of chamber shortening, this can theoretically be performed by manual analysis as well without advanced post-processing software and presents an accessible option for imaging sites that do not have access to the newest software. A topic of further study might be to investigate time expenditure for each analysis method. Future work should assess these analysis techniques in relation to clinical patient status and outcomes and determine their prognostic utility in a larger cohort.

### Study limitations

Our classification of diastolic dysfunction relies on clinical echocardiographic reports performed within four weeks prior to the CMR exam. A change in hemodynamics, i.e., pre- and afterload, can impact diastolic dysfunction grading. A desirable approach in the future would be to conduct echocardiography and CMR on the same day and to compare CMR measurements directly to contemporary echocardiographic quantification of diastolic dysfunction, e.g., by transmitral blood flow and Doppler tissue velocity interrogation. Due to varying RR intervals, AF patients can have a different acquisition triggering scheme than the other subgroups. With prospective triggering late diastole is not imaged; however, in patients with AF, this should not impact measurements significantly as there should not be any change in chamber volume and size during this time due to a lack of atrial kick. Additionally, our sample size was small for the subgroup analysis. Further comparisons between software vendors are required due to differences in tracking algorithms and methodological approaches to contour placement.

## Conclusion

CMR post-processing techniques can assess bi-ventricular and bi-atrial function to monitor atrial dysfunction and myopathy in patients with AF or progressing ventricular diastolic dysfunction. Assessing right and left atrial function with feature tracking or long-axis shortening techniques may allow for early detection of subtle functional abnormalities, even when atrial enlargement is not yet apparent. Our analysis demonstrates that both techniques produce similar results, which indicates that they could potentially be used interchangeably based on the software capabilities of individual sites. Using a CMR-based analysis to understand the individual atrial-ventricular interaction of both the left and right side of the heart in addition to morphology, function, and tissue characteristics allows for a comprehensive interrogation of all four heart chambers. In patients, this could add clinically meaningful information and potentially allow for optimal therapies to be chosen to better target the dysfunction.

### Supplementary Information

Below is the link to the electronic supplementary material.

Supplementary file1 (PDF 396 KB)

## Abbreviations

2CH: Two-chamber
4CH: Four-chamber
AEF: Active emptying fraction
AF: Atrial fibrillation
AUC: Area under the curve
CMR: Cardiovascular magnetic resonance
DD: Diastolic dysfunction
ECV: Extracellular volume
EF: Ejection fraction
FT: Feature-tracking
HFpEF: Heart failure with a preserved ejection fraction
HFrEF: Heart failure with a reduced ejection fraction
LA: Left atrial
LAS: Long-axis shortening
LGE: Late gadolinium enhancement
MAPSE: Mitral annular planar systolic excursion
PEF: Passive emptying fraction
RA: Right atrial
TAPSE: Tricuspid annular planar systolic excursion

## Declarations

### Guarantor

The scientific guarantor of this publication is Kady Fischer.

### Statistics and biometry

One of the authors has significant statistical expertise.

### Study subjects or cohorts overlap

Some study subjects or cohorts have been previously reported in:

• Fischer et al 2018, https://doi.org/10.1186/s12968-018-0446-y

• Guensch et al, 2020, https://doi.org/10.1161/JAHA.119.014739

• Fischer et al, 2022, https://doi.org/10.1161/CIRCHEARTFAILURE.121.008903

### Methodology

*•* Prospective

*•* Observational

*•* Multicenter study
