# Supplementary material for: Analysis of bi-atrial function using CMR feature tracking and long-axis shortening approaches in patients with diastolic dysfunction and atrial fibrillation
Source: Eur Radiol. 2023 May 5;33(10):7226–37. doi: 10.1007/s00330-023-09663-4 (PMC10511591; doi:10.1007/s00330-023-09663-4)
Supplement: Supplementary file 1 — Supplementary file1 (PDF 396 KB) [file 330_2023_9663_MOESM1_ESM.pdf]

SUPPLEMENTAL INFORMATION

Analysis of bi-atrial function using CMR feature tracking and long-axis shortening approaches in patients with diastolic dysfunction and atrial fibrillation

Table of Contents

Supplemental Figure 1: Participant Selection and Dataset Inclusion 2
Supplemental Figure 2: Bland Altman for Reservoir Phase 3
Supplemental Table 1: Patient Characteristics 4
Supplemental Table 2: Bland-Altman Comparisons of Atrial Strain 5
Supplemental Table 3: Correlations Within Diastolic Dysfunction and Atrial Fibrillation 6
Supplemental Table 4: Left Atrial Measurements 7
Supplemental Table 5: Right Atrial Measurements 8

Supplemental Figure 1: Participant Selection and Dataset Inclusion

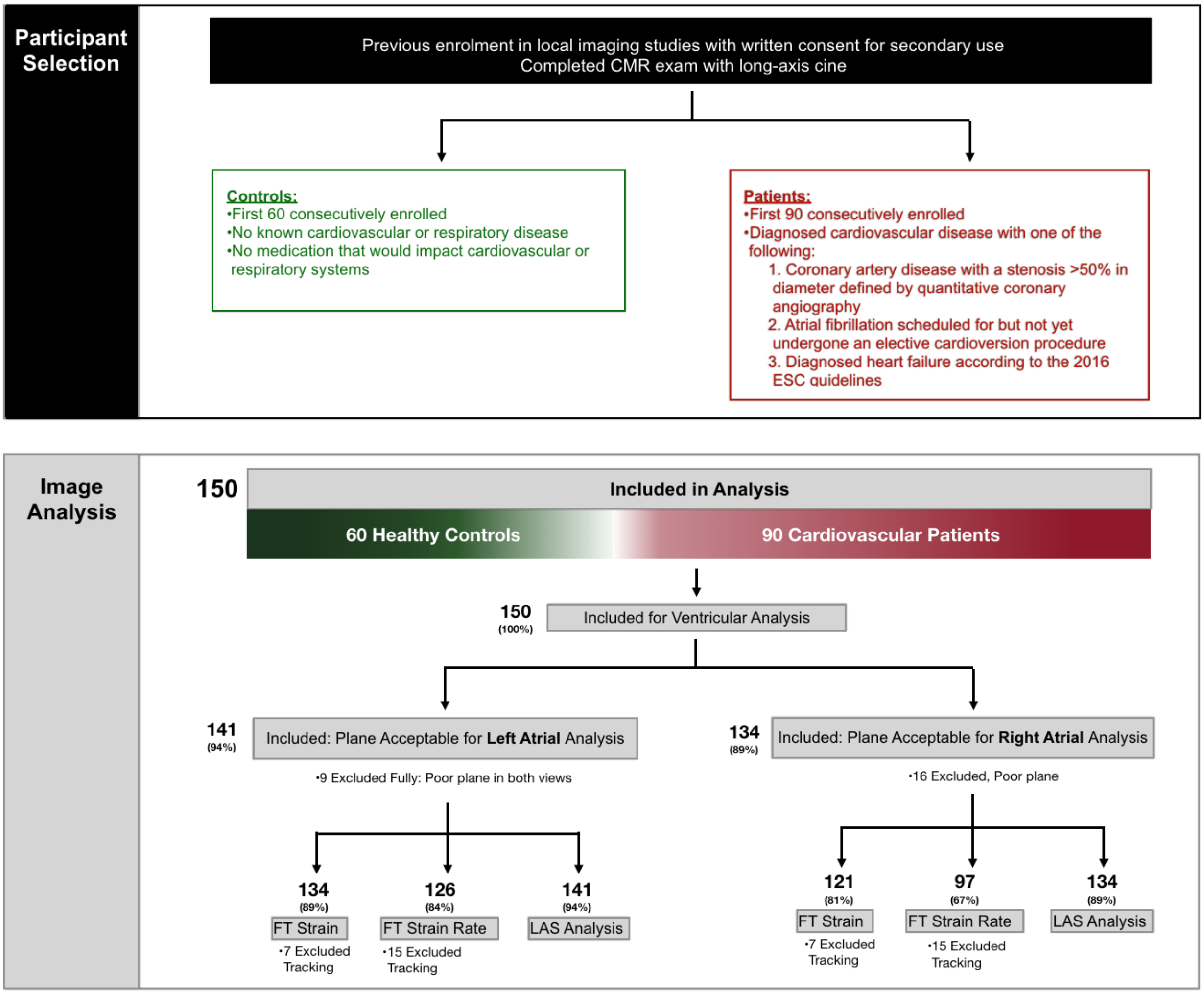

The first 60 healthy controls and first 90 patients enrolled into local imaging research studies who had provided written consent for secondary use of their data were included into the current analysis. Left atrial datasets were excluded when both the 2- and 4-chamber views were unacceptable, while right atrial analysis was dependent on the 4-chamber view only. LAS techniques could analyze 100% of the appropriate planes, while FT techniques had further exclusions due to tracking errors.
FT: Feature tracking, LAS: Long-axis shortening.

Supplemental Figure 2: Bland Altman for Reservoir Phase

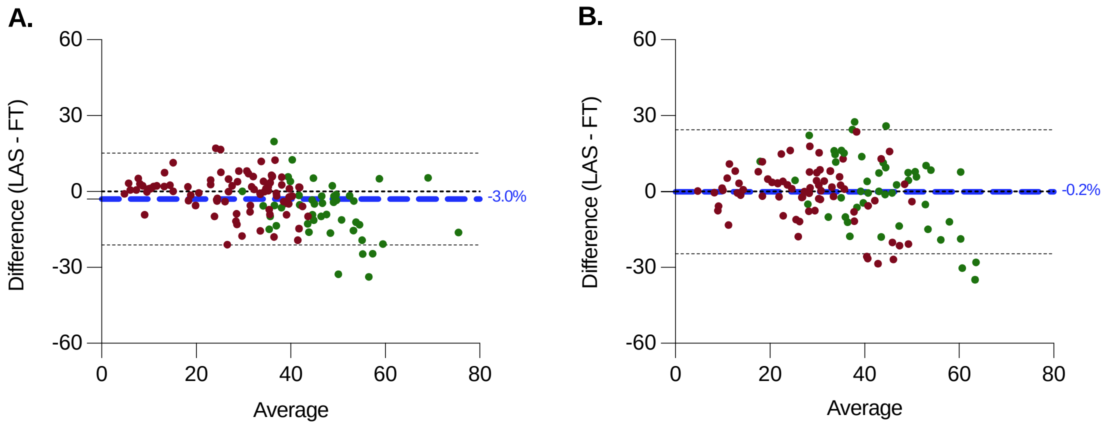

Bias and 95% agreement are shown for the feature tracking (FT) vs long-axis shortening (LAS) measurements of the left (A) and right (B) atrium during the reservoir phase of all participants, patients are highlighted in red, and controls in green.

Supplemental Table 1: Patient Characteristics

	**All Patients** (n=90)	**DD Grade 0** (n=7)	**DD Grade I** (n=24)	**DD Grade II-III (n=20)**	**AF** (n=20)
Age (years)	63±11	61±11	64±10	61±10	65±13
Males	72 (80%)	7 (100%)	16 (67%)	13 (65%)	15 (75%)
Body Mass Index (kg/m^2)	28.7±4.9	30.6±3.3	29.6±5.0	29.5±6.1	28.8±4.4
**Comorbidities**					
Coronary Artery Disease	57 (63%)	7 (100%)	15 (63%)	8 (40%)	8 (40%)
Heart Failure					
• *All diagnosed*	53 (59%)	0	17 (71%)	18 (90%)	13 (65%)
• *LVEF ≥50%*	38 (42%)		12 (50%)	17 (85%)	4 (20%)
• *LVEF <50%*	15 (17%)		5 (21%)	1 (5%)	9 (45%)
Diabetes Mellitus	23 (26%)	2 (29%)	9 (38%)	4 (20%)	5 (25%)
Dyslipidemia	45 (50%)	4 (57%)	15 (63%)	7 (35%)	10 (50%)
**Atrial Fibrillation**					
• *Paroxysmal*					12 (60%)
• *Persistent*					8 (40%)
**Medication**					
ACE Inhibitors	38 (42%)	3 (43%)	11 (46%)	5 (25%)	11 (55%)
Angiotensin II Receptor Blockers	16 (18%)	1 (14%)	6 (25%)	2 (19%)	2 (10%)
Anti-Platelets	58 (64%)	4 (57%)	19 (79%)	11 (55%)	7 (35%)
Beta Blockers	66 (73%)	4 (57%)	18 (75%)	12 (60%)	17 (85%)
Calcium Channel Blockers	12 (13%)	1 (14%)	1 (4%)	3 (15%)	3 (15%)
Diuretics	15 (17%)	1 (14%)	4 (17%)	7 (35%)	2 (10%)
Statins	65 (72%)	6 (86%)	20 (83%)	11 (55%)	10 (50%)

Mean±SD or frequency (percentage of group) are reported. Nineteen patients did not have a recent echocardiography report and thus were not classified to the subgroups. ACE: angiotensin-converting enzyme, AF: atrial fibrillation, DD: diastolic dysfunction, LVEF: left ventricular ejection fraction.

Supplemental Table 2: Bland-Altman Comparisons of Atrial Strain

	**Left Atrium**	**Right Atrium**
Reservoir Phase (%)	-3.0±9.3	-0.2±12.5
Conduit Phase (%)	-1.4±8.7	-0.5±12.4
Booster Phase (%)	-2.4±7.0	0.3±7.1

Bias±SD of long axis extent strain results in comparison to feature tracking of all participants.

Supplemental Table 3: Correlations Within Diastolic Dysfunction and Atrial Fibrillation

	Left Atrium	Right Atrium
Controls	r=0.58, p<0.01	r=0.41, p<0.01
All Diastolic Dysfunction (Grade I-III)	r=0.75, p<0.01	r=0.65, p<0.01
Atrial Fibrillation	r=0.88, p<0.01	r=0.73, p<0.01

Correlation coefficients (r) compare the atrial reservoir (peak) strain measurements between the feature tracking strain analysis, and the long-axis shortening techniques.

Supplemental Table 4: Left Atrial Measurements

	Controls	All Patients	Controls Oldest Tertile	DD Grade 0	DD Grade I	DD Grade II-III	AF
Maximum Volume Index (ml/m^2)	44±10	42±16	48±12	38±7#	38±13#	45±18	48±18
Maximum Area Index (cm/m^2)	13±2	12±4	14±3	11±2#	12±3#	13±4	14±4
**LA Reservoir**							
Total Emptying Volume (ml)	53±14	40±15*	56±16	50±10	45±17	36±13#	29±14#
LA Ejection Fraction (%)	64±6	49±16*	62±6	61±7	59±9	43±13#	31±14#
LAS Reservoir Strain (%)	42±8	25±11*	37±9	33±5	30±9#	22±11#	15±11#
LAS Reservoir ΔDistance (mm)	15±3	10±5*	16±3	13±4	12±5#	9±4#	6±4#
FT Reservoir Strain (%)	48±12	26±13*	43±12	32±8	30±10#	24±13#	14±11#
FT Reservoir Strain Rate (/s)	2.3±1.0	1.0±0.6*	1.9±0.7	1.6±0.2	1.5±0.5	1.4±0.6	1.1±0.6#
**LA Conduit**							
Passive Emptying Volume (ml)	32±10	21±10*	27±10	21±2	21±10	18±9#	26±12
Passive Emptying Fraction (%)	39±10	26±9	30±8	26±5	28±9	24±9#	28±10
LAS Conduit Strain (%)	27±9	15±8*	22±6	16±4	17±7	13±10#	13±8#
FT Conduit Strain (%)	32±11	17±7*	23±10	15±6	18±9	13±8#	12±8#
FT Conduit Strain Rate (/s)	-3.7±1.8	-1.3±0.6*	-2.4±0.9	-1.3±0.4#	-1.3±0.6#	-1.3±0.5#	-1.0±0.4#
**LA Booster**							
Active Emptying Volume (ml)	21±10	22±9	29±11	29±9	24±10	15±6#	-
Active Emptying Fraction (%)	40±8	39±13	45±8	47±10	42±10	28±13#	-
LAS Active Strain (%)	15±5	14±6	15±4	16±4	15±5#	11±7	-
FT Active Strain (%)	17±5	17±7	20±5	20±5	16±7	14±7#	-
FT Active Strain Rate (/s)	-2.4±0.9	-2.0±0.9	-2.6±0.8	-2.3±0.7	-2.1±0.9	-1.8±1.1	-

Mean±SD. AF: atrial fibrillation, DD: diastolic dysfunction, FT: feature-tracking, LA: left atrial, LAS: long-axis shortening,
*p<0.05 all patients vs. control population statistically correcting for age.
#p<0.05 and oldest tertile of controls compared to cardiovascular patient subgroups accounting for multiple comparisons.

Supplemental Table 5: Right Atrial Measurements

	Controls	All Patients	Controls Oldest Tertile	DD Grade 0	DD Grade I	DD Grade II-III	AF
Maximum Volume Index (ml/m^2)	49±12	42±15	53±12	40±12	36±11$^{\#}$	41±17$^{\#}$	47±15
Maximum Area Index (cm/m^2)	13±2	13±5	14±2	14±9	11±2$^{\#}$	12±3$^{\#}$	14±5
**RA Reservoir**							
Total Emptying Volume (ml)	48±14	38±15*	50±12	43±14	37±20	33±15$^{\#}$	38±11$^{\#}$
RA Ejection Fraction (%)	52±8	45±12*	50±6	51±10	49±9	42±14	40±13$^{\#}$
LAS Reservoir Strain (%)	42±10	27±12*	39±10	36±10	34±10	25±10$^{\#}$	18±11$^{\#}$
LAS Reservoir ΔDistance (mm)	16±3	11±6*	16±3	15±3	13±5	10±5$^{\#}$	7±7$^{\#}$
FT Reservoir Strain (%)	42±15	28±15*	41±16	31±9	35±12	22±10$^{\#}$	16±8$^{\#}$
FT Reservoir Strain Rate (/s)	2.4±1.1	1.7±0.8*	2.4±1.3	1.9±0.8	2.1±0.9	1.5±0.5	1.2±0.7
**RA Conduit**							
Passive Emptying Volume (ml)	27±10	17±12	22±8	24±5	18±16	14±10$^{\#}$	35±14
Passive Emptying Fraction (%)	30±8	20±9	22±7	23±4	21±10	18±11	36±14
LAS Conduit Strain (%)	29±9	17±9*	24±8	23±16	19±8	13±7$^{\#}$	16±10$^{\#}$
FT Conduit Strain (%)	28±12	19±11	26±13	17±4	25±11	13±10$^{\#}$	14±7$^{\#}$
FT Conduit Strain Rate (/s)	-2.7±1.4	-1.4±0.8	-1.9±0.9	-1.2±0.2$^{\#}$	-1.8±0.9	-1.0±0.4$^{\#}$	-1.1±0.6
**RA Booster**							
Active Emptying Volume (ml)	21±11	22±10	29±10	29±16	21±10	21±12	-
Active Emptying Fraction (%)	30±9	34±12	36±7	36±21	36±11	32±14	-
LAS Active Strain (%)	14±6	16±6	16±8	16±4	18±7	14±7	-
FT Active Strain (%)	14±5	14±8	16±5	17±6	18±9	11±5$^{\#}$	-
FT Active Strain Rate (/s)	-1.7±0.6	-1.4±0.9	-1.8±0.4	-2.1±0.8	-2.2±0.9	-1.5±0.6	-

Mean±SD. AF: atrial fibrillation, DD: diastolic dysfunction, FT: feature-tracking, LAS: long-axis shortening, RA: right atrial,
*p<0.05 all patients vs control population statistically correcting for age.
$^{\#}$p<0.05 and oldest tertile of controls compared to cardiovascular patient subgroups accounting for multiple comparisons.
